# Experimental Study on Double-Sided Chemical Mechanical Polishing of Molybdenum Substrates for LED Devices

**DOI:** 10.3390/mi17020150

**Published:** 2026-01-23

**Authors:** Zhihao Zhou, Jiabin Wang, Zhongwei Hu, Pinhui Hsieh, Xipeng Xu

**Affiliations:** 1 Institute of Manufacturing Engineering, Huaqiao University, Xiamen 361021, China; alan.zhou@sanan-e.com (Z.Z.); vv_3020@163.com (J.W.); xpxu@hqu.edu.cn (X.X.); 2 State Key Laboratory for High Performance Tools, Huaqiao University, Xiamen 361021, China; 3 Fujian Jing’An Opto. Electronics Co., Ltd., Quanzhou 362411, China

**Keywords:** molybdenum substrate, double-sided chemical mechanical polishing, orthogonal experiment, process parameter optimization, surface quality

## Abstract

As LED devices continue to advance toward miniaturization and higher power density, heat dissipation has become a critical factor constraining their reliability and service life. Molybdenum is widely employed as a substrate material in LED devices owing to its high thermal conductivity and low coefficient of thermal expansion. However, substrate applications impose stringent requirements on surface finish, flatness, and low-damage processing. Chemical mechanical polishing (CMP) can effectively balance global and local flatness and serves as the final step in producing high-quality molybdenum substrate surfaces. To enable efficient and precise processing of molybdenum substrates, this study adopts an orthogonal experimental design for double-sided CMP to systematically investigate the effects of polishing pressure, polishing slurry pH, additives in the polishing slurry, and abrasive particle size on the material removal rate (MRR) and surface roughness (Sa). An optimal parameter combination was identified via weight-matrix optimization: a polishing pressure of 115 kPa, pH 11, H_2_O_2_ (0.5%) and glycine (5 mg/L) as additives, and an abrasive particle size of 0.6 μm. Under these conditions, the MRR reached 80 nm·min^−1^ and Sa decreased to 1.1 nm, yielding a smooth, mirror-like surface. The results indicate that multi-factor synergistic optimization can substantially enhance both surface quality and processing efficiency in double-sided CMP of molybdenum substrates, providing a process basis for applications in high-power LED devices.

## 1. Introduction

Light-emitting diodes (LEDs) represent a new generation of lighting sources and offer high luminous efficiency, low energy consumption, fast response, and long service life [1]. As device structures continue to miniaturize and evolve toward higher power densities [2,3], higher operating currents are commonly required to achieve increased brightness, which elevates the junction temperature and promotes thermal-stress accumulation. According to electronic design rules, the average lifetime is reduced by 50% for every 10 °C increase in temperature [4,5]. Consequently, effective heat dissipation has become a critical issue for improving the reliability of high-power LEDs. Traditional LED devices often employ silicon wafers as substrates; however, silicon may be unable to meet heat-dissipation requirements under high-power conditions, leading to a pronounced temperature rise [6]. Although high-thermal-conductivity substrate materials such as AlN, Si_3_N_4_, CuW, MoCu, and diamond-based materials exhibit excellent thermal matching and heat-dissipation performance [7,8,9,10], their high manufacturing and processing costs limit their large-scale adoption. Therefore, molybdenum substrates have attracted increasing attention in industry [11,12]. Pure molybdenum features high thermal conductivity (approximately 138 W·m^−1^·K^−1^) and a low coefficient of thermal expansion (4.9 × 10^−6^ K^−1^). It also has a high melting point (2623 °C) and good high-temperature stability [13,14]. These properties confer potential advantages to molybdenum in heat-dissipation substrates for high-power devices [15].

To meet the stringent requirements of LED molybdenum substrates for surface flatness and finish, the molybdenum plate’s raw materials must undergo planarization steps such as grinding and polishing to obtain a mirror-like surface while minimizing defects, including scratches, pits, and spots. As the final manufacturing stage, the polishing quality is decisive in determining whether molybdenum substrates can satisfy LED application requirements [16]. Chemical mechanical polishing (CMP), a critical process in semiconductor manufacturing [17], provides a practical route to achieving this goal. In CMP, the polishing slurry chemistry, abrasive characteristics, polishing pad, and operating conditions act in a strongly coupled manner; material removal fundamentally arises from the synergy between slurry-induced chemical corrosion and the mechanical action of abrasives and the polishing pad. Consequently, CMP has become an important technique for producing ultra-smooth, low-damage surfaces on molybdenum substrates [18].

Most studies aimed at improving the surface quality and processing efficiency of molybdenum by chemical mechanical polishing (CMP) have focused on the polishing slurry formulation [19,20,21,22] and the associated removal mechanisms [23,24]. Heon-Yul Ryu et al. [19] investigated the influence of H_2_O_2_ on the static corrosion rate (SER) and removal rate (RR) of molybdenum under different pH conditions and reported that the SER increased with increasing H_2_O_2_ concentration; at pH = 2, a high RR of 88 nm/min and a low surface roughness of 2.04 nm were achieved. He et al. [20] performed CMP using KIO_3_ as the oxidant and colloidal SiO_2_ as the abrasive, showing that the RR increased while the SER decreased in acidic solutions; at pH = 2, a thin film that is more readily removed by mechanical action could form, resulting in a smoother surface. Choi et al. [21] compared the adsorption mechanisms and stability of glycine and benzotriazole (BTA) on Mo surfaces, showing that at a lower concentration (30 mM), glycine provided stronger inhibition through rapid electrostatic interactions, whereas at a higher concentration (100 mM), BTA exhibited markedly enhanced inhibition due to the formation of more stable coordination with molybdenum atoms. Wu et al. [22] further examined the combined use of a nonionic surfactant and BTA, demonstrating that their combination enhances surface coverage and shielding on molybdenum, reduces the SER, and improves the surface chemical state. In terms of material removal, Panagiotis et al. [23] compared different abrasives and process parameters in molybdenum polishing and noted that the MRR was strongly affected by abrasive concentration; at 6 wt% alumina, a higher MRR was obtained, and an alkaline slurry was suggested to be more favorable for improving removal efficiency in their system. Yan Qiusheng et al. [24] compared three abrasives (W_3_SiC, α-Al_2_O_3_, and CeO_2_) for molybdenum wafers using single-sided free grinding. They found that SiC and Al_2_O_3_, which are substantially harder than molybdenum, can achieve higher removal rates but are more likely to introduce disordered scratches and pits. By contrast, CeO_2_ improves surface quality at the cost of a lower removal rate. For LED applications, double-sided CMP serves as the final planarization step for molybdenum substrates. The finished surface must meet stringent requirements, including flatness, smoothness, a mirror finish, and low defects (no scratches, no pits, and no spots). However, research specifically addressing the double-sided CMP process for molybdenum substrates remains limited. To enable high-quality and high-efficiency double-sided CMP for LED molybdenum substrates, this study selects polishing pressure, polishing slurry pH, additive system, and abrasive particle size as the key process factors for experimental investigation.

Although existing studies on molybdenum CMP have largely emphasized chemical reaction mechanisms, reports addressing ultra-precision machining processes for device-substrate applications remain limited. Accordingly, this work conducts orthogonal experiments on double-sided CMP of molybdenum substrates using precision polishing equipment. The effects of polishing pressure, polishing slurry pH, additive system, and abrasive particle size on the MRR and Sa are comprehensively investigated. An optimal process combination suitable for industrial applications is then determined using a weight-matrix optimization algorithm, enabling high-efficiency and high-quality precision machining of molybdenum substrate surfaces.

## 2. Double-Sided Chemical Mechanical Polishing Experiments

### 2.1. Surface Morphology and Formation Mechanism

**Figure 1:**
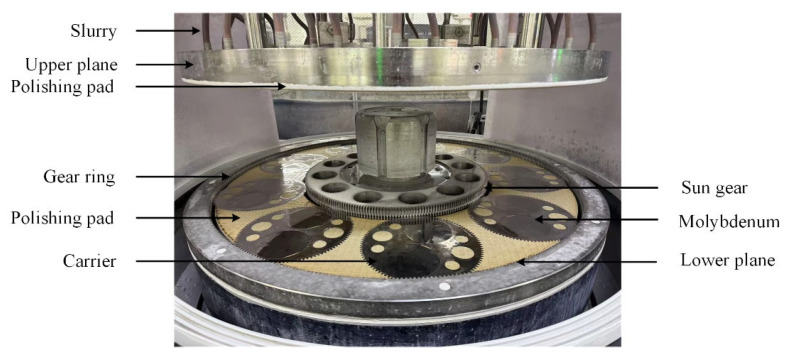
Double-sided chemical mechanical polishing system.

**Figure 2:**
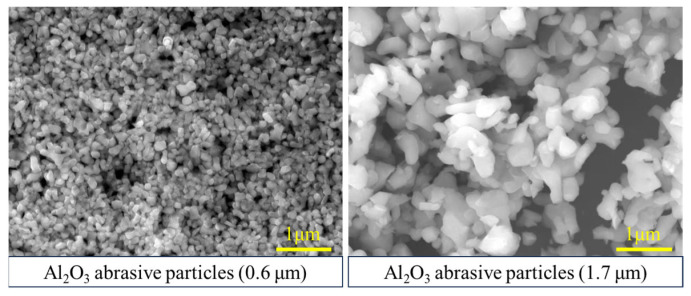
Microstructure of Al_2_O_3_ abrasive particles.

Polishing experiments were performed using a 13BF-3M9L-M precision double-sided planar polishing machine (Suzhou Bohongyuan Equipment Co., Ltd., Suzhou, China). The system is driven by three motors, including a 7.5 kW lower-plane motor, a 4.9 kW upper-plane motor, and a 1.5 kW sun-gear motor, as shown in Figure 1. Both the upper and lower polishing pads feature a grid-groove structure, with an outer diameter of 950 mm, an inner diameter of 560 mm, and a thickness of 50 mm. During polishing, the upper plane and lower plane rotated at 3.3 rpm and 10 rpm, respectively. The slurry was delivered to the polishing zone through the upper supply pipe using a pump at a volumetric flow rate of 8–10 LPM. The polishing slurry’s abrasive particles were Al_2_O_3_ with particle sizes of 0.6 μm and 1.7 μm, as shown in Figure 2. The polishing slurry was prepared by mixing the stock solution and deionized water at a 1:1 volume ratio. A 35–37% H_2_O_2_ solution was used as the oxidant, and glycine (>99%) was used as the inhibitor.

**Figure 3:**
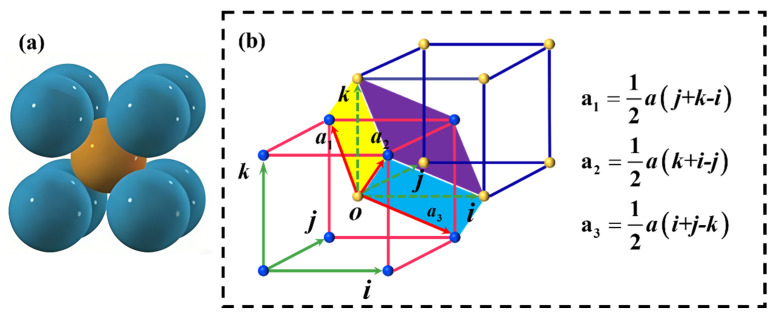
Schematic diagram of the lattice type of molybdenum. (**a**) Molybdenum crystal cell structure; (**b**) body-centered cubic primitive cell.

The workpiece was a molybdenum substrate with a diameter of 4 inches and a thickness of 150 μm. Molybdenum is a transition-metal polycrystalline material with an A2-type body-centered cubic structure at room temperature and a lattice constant of 3.14 Å. Pure molybdenum exhibits strong atomic bonding; its plasticity is lower than that of face-centered cubic aluminum but higher than that of tungsten. It also has a high elastic modulus, good high-temperature mechanical stability, and certain toughness and wear resistance, resulting in removal and damage behaviors that differ from those of typical brittle semiconductor materials during processing. The physical properties of molybdenum are summarized in Table 1, and a schematic of the molybdenum lattice structure is provided in Figure 3.

The MRR of the molybdenum substrate is derived from the mass change before and after polishing, and the calculation formula is as follows:

(1) $$MRR=\frac{\left(m_{0}-m_{1}\right)\times 10^{7}}{\rho \times S\times t}$$

In the formula, MRR is the material removal rate/(nm·min^−1^); *m*_0_ is the mass of the molybdenum substrate before polishing/g; *m*_1_ is the mass of the molybdenum substrate after polishing/g; *ρ* the density of the molybdenum wafer/(g·cm^3^); *S* is the area of the molybdenum wafer/cm^2^; and *t* is the polishing time/min. The mass of the molybdenum substrate was weighed using an electronic balance, and the average of three weighings was taken after polishing each *m*_0_ and *m*_1_. The surface roughness Sa was measured using a three-dimensional optical profiler. Surface roughness Sa and surface morphology were measured using the Bruker contour 3D optical profilometer. Surface pitting defects were observed using an Axia ChemiSEM HiVac scanning electron microscope (Thermo Fisher Scientific Inc., Waltham, MA, USA).

### 2.2. Orthogonal Experiment

An *L*16 (4^3^ × 2^6^) hybrid orthogonal array was used to conduct double-sided CMP experiments on molybdenum substrates, effectively reducing the number of experiments while ensuring factor-level coverage and representative combination. The experimental scheme and measurement results are shown in Table 2 and Table 3, respectively. All experiments were conducted in a cleanroom laboratory with the ambient temperature controlled within room temperature to minimize the impact of external contamination on the test results.

The orthogonal experimental parameters used in this study included material removal rate (nm/min^−1^), surface roughness (nm), and surface defects (SD). After polishing, the surface of the molybdenum substrate will produce surface pit defects. Pits were observed by a scanning electron microscope. Surfaces that had pits with radii greater than or equal to the abrasive particle size were recorded as having “Pits”, and they were recorded as “OK” otherwise.

## 3. Results and Discussion

### 3.1. Analysis of Material Removal Process

**Figure 4:**
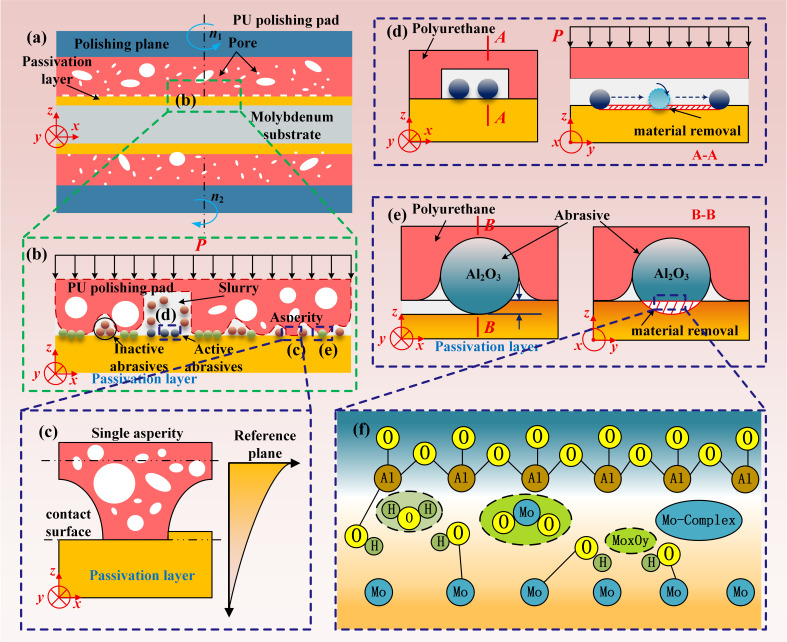
Material removal process of double-sided chemical mechanical polishing (CMP) on molybdenum wafers: (**a**) full process; (**b**) different contact conditions; (**c**) single-roughness-peak contact; (**d**) three-body removal; (**e**) two-body removal; (**f**) chemical bonding.

Chemical mechanical polishing (CMP) achieves material removal and planarization through the combined action of (i) chemical corrosion and surface softening induced by reagents in the polishing slurry and (ii) mechanical abrasion generated by abrasive particles. In this study, a grid-grooved polyurethane polishing pad was employed (Figure 4a). Several representative contact states and removal modes during double-sided CMP of the molybdenum substrate are schematically illustrated in Figure 4b. Single roughness peak–molybdenum substrate contact: As shown in Figure 4c, the single roughness peak on the polyurethane polishing pad contacts the passivation layer on the molybdenum substrate surface, achieving material removal. Molybdenum substrate surface removal under single-roughness-peak contact is a purely mechanical removal process without abrasive particles. Abrasive–molybdenum substrate three-body removal: As shown in Figure 4d, considering the large product of the diameter and polishing speed of the double-sided polishing machine, the polishing slurry flows rapidly along the grooves in the grid-type polyurethane polishing pad under centrifugal motion. Therefore, the three-body removal formed by the erosion effect of the abrasive particles on the molybdenum substrate surface cannot be ignored. Abrasive–molybdenum substrate two-body removal: As shown in Figure 4e, due to the deformation of the rough peaks of the polishing pad, when the polishing slurry is sufficient, the abrasive particles in the polishing slurry will be embedded in the rough peaks under the action of polishing pressure. As the polishing disk rotates, the passivation layer surface of the molybdenum substrate is mechanically removed by two-body wear.

From the perspective of chemical action, the polishing slurry system undergoes redox reactions and dissolution–complexation processes at the molybdenum surface (Figure 4f). Chemical reagents in the slurry react with molybdenum via oxidation and subsequent transformation, softening the surface, accelerating material removal, and mitigating surface damage. The reaction sequence is summarized as follows. Under the action of H_2_O_2_, Mo is first oxidized to MoO_2_ and subsequently forms higher oxides such as Mo_2_O_5_ and MoO_3_ [25,26]. These oxides react with water to form metamolybdic acid, which further converts to molybdate species under alkaline conditions:

(2) $$Mo+H_{2}O_{2}\to MoO_{2}+\;H_{2}$$

(3) $$2MoO_{2}+\;H_{2}O_{2}\to \;Mo_{2}O_{5}+\;H_{2}O$$

(4) $$Mo_{2}O_{5}+\;H_{2}O_{2}\to \;2MoO_{3}+H_{2}O$$

(5) $$MoO_{3}+\;H_{2}O\;\to H_{2}MoO_{4}$$

(6) $$H_{2}MoO_{4}+\;2OH^{-}\to 2H_{2}O\;+\;MoO_{4}^{2-}$$

(7) $$MoO_{3}+\;2OH^{-}\to \;MoO_{4}^{2-}+\;H_{2}O$$

Glycine further contributes through dissociation and coordination in the slurry. By forming complexes within the oxide layer on metallic molybdenum, glycine suppresses excessive corrosion and supports stable, controllable removal:

(8) $$H_{3}NCH_{2}COOH\;\to \;H_{3}NCH_{2}COO-\;\to \;H_{2}NCH_{2}COO^{-}$$

(9) $$\begin{array}{l} MoO_{3}+\;2H_{3}NCH_{2}COO^{-}+\;2NH_{2}CH_{2}COO^{-}\to \\ \;MoO_{3}{\left(H3NCH2COO^{-}\right)}_{2}+\;{\left(NH_{2}CH_{2}COO^{-}\right)}_{2} \end{array}$$

These complexes cover and passivate active sites, inhibiting localized rapid dissolution. Simultaneously, abrasive cutting continuously removes the oxide and coordination layers, exposing fresh surfaces and sustaining a cyclic process of generation–removal–regeneration. Through this coupling of chemical modification and mechanical abrasion, CMP achieves chemical–mechanical synergy and stable material removal.

### 3.2. Effect on Material Removal Rate

#### 3.2.1. Range Analysis of the MRR

Orthogonal experiments are characterized by orthogonality and comprehensive comparability. For a given factor, the mean response value ki of an experimental index at each level can be treated as an approximation of the factor’s main effect at that level. Accordingly, the range RA = max{*ki*} − min{*ki*} is used to quantify the magnitude of a factor’s influence on response fluctuations. A larger R value indicates a more pronounced change in the response attributable to that factor and, therefore, a greater contribution to the experimental outcome.

**Figure 5:**
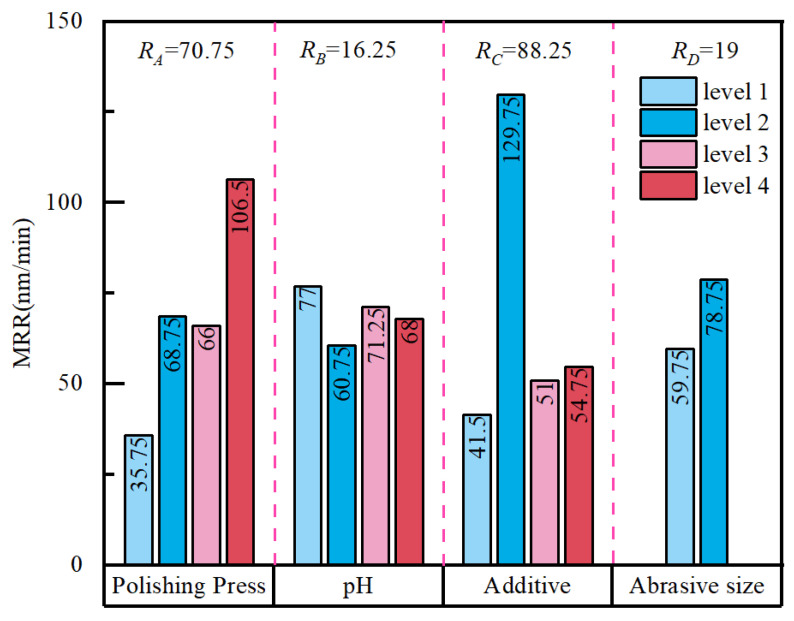
Effects of various factors on material removal rate.

Based on the mean MRR values at each factor level, the MRR trend plot in Figure 5 enables direct comparison of factor influence. The range analysis shows that the additive system (C) exerts the strongest effect (*R_C_* = 88.25), followed by polishing pressure (*A*) (*R_A_* = 70.75), whereas abrasive particle size (*D*) and pH (*B*) have comparatively small effects (*R_D_* = 19, *R_B_* = 16.25). Thus, the dominant factors affecting the MRR are ranked as *C* > *A* > *D* > *B*.

In terms of level effects, the additive system is the primary determinant of the MRR. When only H_2_O_2_ is added, the MRR increases markedly and reaches its highest level, indicating that the oxidant accelerates oxidation on the molybdenum surface and promotes formation of a reaction layer that is more readily removed by mechanical shear, thereby substantially enhancing overall removal efficiency. By contrast, when H_2_O_2_ is combined with glycine, the inhibitory effect of glycine reduces surface reaction activation and suppresses localized rapid dissolution. As a result, the MRR decreases while providing a process basis for subsequent improvements in surface-quality indicators. Polishing pressure also shows a pronounced positive effect: the MRR generally increases with increasing pressure, consistent with the Preston relationship. This behavior suggests that, at constant relative velocity, higher pressure intensifies real interfacial contact and shear, thereby strengthening the removal capability of abrasives and polishing-pad asperities acting on the reaction layer. Moreover, faster surface renewal under higher pressure can indirectly facilitate continued chemical reactions, further increasing the MRR. The effect of abrasive particle size on the MRR is less significant than that of pressure, yet it remains positive. Increasing the particle size from 0.6 μm to 1.7 μm raises the MRR from 59.75 nm·min^−1^ to 78.75 nm·min^−1^, implying that a greater cutting contribution from individual abrasive particles is beneficial for removal under these conditions. However, potential impacts on defects and roughness must be assessed together with subsequent Sa and SD measurements. In contrast, pH exhibits the weakest main effect on the MRR. Under weakly acidic conditions (pH = 4), the MRR is slightly higher than under neutral and alkaline conditions, but the difference remains limited within the present slurry formulation and pressure range, indicating that pH primarily plays a tuning role rather than serving as a dominant factor in this system.

#### 3.2.2. Analysis of Variance (ANOVA) for the MRR

Analysis of variance (ANOVA) examines the relationship between categorical factors and a continuous response variable. It evaluates whether differences among the overall means at different levels of a given factor are statistically significant by comparing the variation attributable to that factor with the variation attributable to experimental error. In this work, the total sum of squares (*S_T_*) and the error sum of squares (*S_E_*) were calculated accordingly. The computation procedure is illustrated below, using factor *A* as an example:

(10) $${C=K}^{2}/n$$

(11) $$S_{T}=\sum_{i=1}^{n}y_{i}-C$$

(12) $$S_{A}=\sum_{i=1}^{r}n_{i}•{\bar{K}}_{\mathrm{Ai}}^{2}-C$$

(13) $$S_{E}{=S}_{T}-S_{A}-S_{B}-S_{C}$$

(14) $$F_{A}{=S}_{A}/S_{E}$$

Here, *C* denotes a standard constant, *K* represents the sum of squares, *S_A_* is the mean square of factor *A*, and *r* is the number of levels. The F-statistic for factor *A* is denoted as *F_A_*. When a blank column exists in the orthogonal array, the error sum of squares is obtained by subtracting the sums of squares of all factors from the total sum of squares. Conversely, when all columns of the orthogonal array are fully utilized (i.e., each column is assigned), the error sum of squares is taken as the smallest among the sums of squares of the factors.

Table 4 summarizes the ANOVA results for the MRR. Additives (*C*) and polishing pressure (*A*) contributed 57.07% and 28.92% to the MRR, respectively, confirming their dominant influence. Abrasive particle size (*D*) accounted for 12.43%, whereas pH (*B*) contributed only 1.58%. At the significance level α = 0.05, the F values for factors *C* and *A* were significant, while those for *D* and *B* were not. This indicates that, within the investigated experimental range, the MRR is primarily governed by the synergistic interplay between chemical oxidation and mechanical removal, whereas pH and particle size exert only secondary, modulating effects. Overall, the material removal behavior is dominated by the chemical oxidation–mechanical removal synergy, and improving removal efficiency therefore depends chiefly on optimizing the additive type and concentration and selecting an appropriate polishing pressure.

### 3.3. Effect on Surface Roughness

#### 3.3.1. Range Analysis of Sa

**Figure 6:**
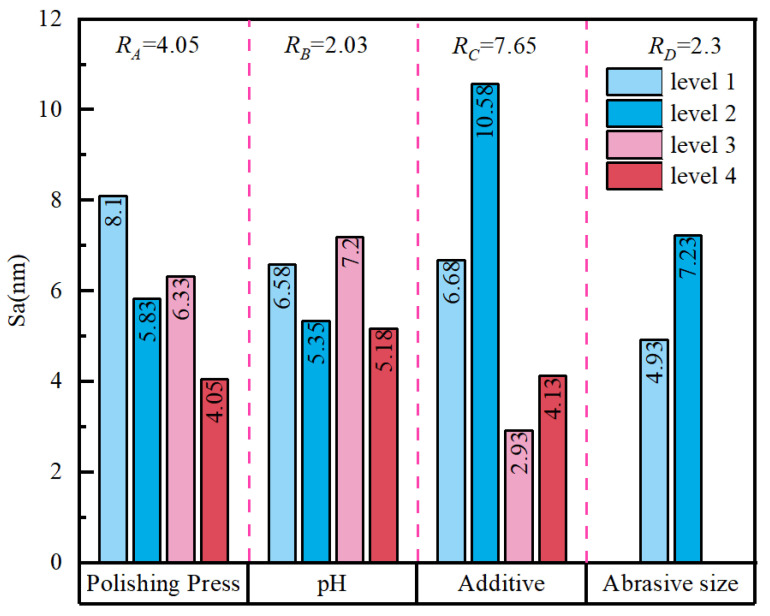
Effects of various factors on surface roughness.

The main effects on Sa were evaluated using range analysis, and the results are presented in Figure 6. The calculated ranges were *R_C_* = 7.65, *R_A_* = 4.05, *R_D_* = 2.30, and *R_B_* = 2.03. These values indicate that, within the investigated parameter window, the additive system was the dominant factor affecting Sa, followed by polishing pressure and abrasive particle size, whereas pH exerted the weakest influence. When the mean responses at each level are considered, the chemical formulation emerges as the primary determinant of surface quality. Sa was generally higher when only H_2_O_2_ was used, and pit defects were more likely to occur. After introducing glycine, Sa decreased markedly, and SD shifted from “Pits” to “OK”. This trend is clearly reflected in the comparative experiments. For example, the seventh, eighth, and ninth groups of experiments, conducted with the compound additive system, yielded Sa values of approximately 2.1, 1.6, and 1.5 nm, respectively, and all were assessed as “OK”. In contrast, the 5th, 11th, 12th, and 15th groups of experiments, performed under conditions involving only H_2_O_2_ or lacking effective suppression and control, produced Sa values of approximately 13.2, 9.6, 9.9, and 10.5 nm, respectively, and all exhibited pits. These observations suggest that, in a strongly oxidizing environment, the absence of inhibitory/complexing components to homogenize and regulate reaction and dissolution processes makes the formation and removal of the surface reaction layer more susceptible to spatial heterogeneity, thereby promoting local over-corrosion and defect amplification.

From a defect-formation standpoint, “Pits” are often associated with local indentation, slippage, and pull-out of abrasive grains under an external load. Higher polishing pressure increases interfacial contact stress, while larger abrasive grains impose a greater single-grain load distribution and a larger indentation scale. The combined effect of these factors increases the likelihood of localized stress concentration, thereby elevating the risk of pitting and roughness deterioration. By contrast, glycine buffers the oxidation–dissolution process on the molybdenum surface through adsorption and complexation, helping to suppress localized runaway of static corrosion and to stabilize the uniformity of the reaction layer. This promotes the formation of a thinner oxide/complex layer that is more readily sheared off, reducing cutting fluctuations and surface morphology variability during polishing. Consequently, Sa decreases overall and defect occurrence is reduced.

#### 3.3.2. Analysis of Variance (ANOVA) for Sa

Table 5 summarizes the ANOVA results for surface Sa. Based on the sums of squares and contribution ratios, the additive system (*C*) exerts the strongest influence on Sa, contributing 50.35% with an F-value of 6.31, confirming that the slurry formulation is the primary source of Sa variation. Abrasive particle size (*D*) and polishing pressure (*A*) contribute 29.05% and 16.14%, respectively (F = 3.64 and 2.02), indicating clear secondary effects. These factors mainly modulate surface waviness and defect sensitivity by altering local interfacial contact stress, abrasive indentation scale, and the morphology of shear-driven removal. In contrast, pH (*B*) shows the weakest influence, with a contribution of 4.45% and an F-value of 0.56, and primarily serves as an auxiliary regulator of the reaction rate and film stability. Mechanistically, Sa reduction relies principally on the coupled oxidation–inhibition/complexation regulation provided by the H_2_O_2_–glycine system, which promotes a more uniform reaction layer and more controllable removal, thereby suppressing micro-topographical fluctuations. By comparison, defects such as pits are dominated by mechanical effects, particularly local stress concentrations associated with abrasive particle size and polishing pressure. Therefore, adopting a glycine-containing compound additive system as the chemical basis, while prioritizing smaller abrasive particles and maintaining polishing pressure at a moderate level, can reduce indentation-related defect risk while preserving the required material removal capability. This interpretation is consistent with literature reports on the H_2_O_2_-driven oxidation–dissolution mechanism and the inhibition/adsorption behavior of glycine on molybdenum surfaces [27], and it provides statistical and mechanistic support for subsequent weight-matrix optimization and process-window determination.

### 3.4. Process Parameter Optimization Based on Weight-Matrix Method

To achieve synergistic optimization of the MRR and Sa, this study adopts the orthogonal experimental design matrix analysis method proposed by Zhou [28] to comprehensively evaluate and optimize the two indices. In this approach, orthogonal experimental data are mapped into a three-layer structural model, and the influence weight of each factor and level on the target index is calculated. The weight-matrix formulation is expressed as follows:

(15) ω=MKS

(16) $$\omega^{T}=\left[\omega_{1},\omega_{2},\ldots ,\omega_{m}\right]$$

where *M*, *K*, and *S* denote the matrices for the experimental index layer, factor layer, and level layer, respectively. ω_1_ represents the influencing index (ω_2_ denotes the weight of a second-layer factor with respect to the first-layer influencing index, and so forth).

Accordingly, the weight vectors for the MRR and Sa are calculated separately. The overall weight matrix for the orthogonal experimental indices is then taken as the average of the weight matrices of the individual indices, as given by

(17) $$\omega =\left(\omega_{MRR}+\omega_{Sa}\right)/2=\left[\begin{array}{r} 0.0458\\ 0.0762\\ 0.0719\\ 0.1146\\ 0.0259\\ 0.0268\\ 0.0238\\ 0.0285\\ 0.0772\\ 0.1336\\ 0.1403\\ 0.1147\\ 0.0638\\ 0.0569 \end{array}\right]=\left[\begin{array}{l} A_{1}\\ A_{2}\\ A_{3}\\ A_{4}\\ B_{1}\\ B_{2}\\ B_{3}\\ B_{4}\\ C_{1}\\ C_{2}\\ C_{3}\\ C_{4}\\ D_{1}\\ D_{2} \end{array}\right]$$

The comprehensive weighting results indicate that the influence weights corresponding to the four polishing-pressure levels (*A*) are *A*1 = 0.0458, *A*2 = 0.0762, *A*3 = 0.0719, and *A*4 = 0.1146. Considering both the MRR and Sa, *A*4 yields the largest weight, indicating that a higher polishing pressure is more favorable for achieving the optimal overall performance. Similarly, *B*4 has the largest weight for the pH (*B*), *C*3 has the largest weight for the additive (*C*), and *D*1 has the largest weight for the abrasive particle size (*D*). Therefore, the optimal comprehensive parameter combination is *A*4*B*4*C*3*D*1. This outcome also indicates the overall factor influence ranking on the comprehensive performance: additives exert the greatest effect, followed by polishing pressure and then abrasive particle size, while pH has the least effect.

**Figure 7:**
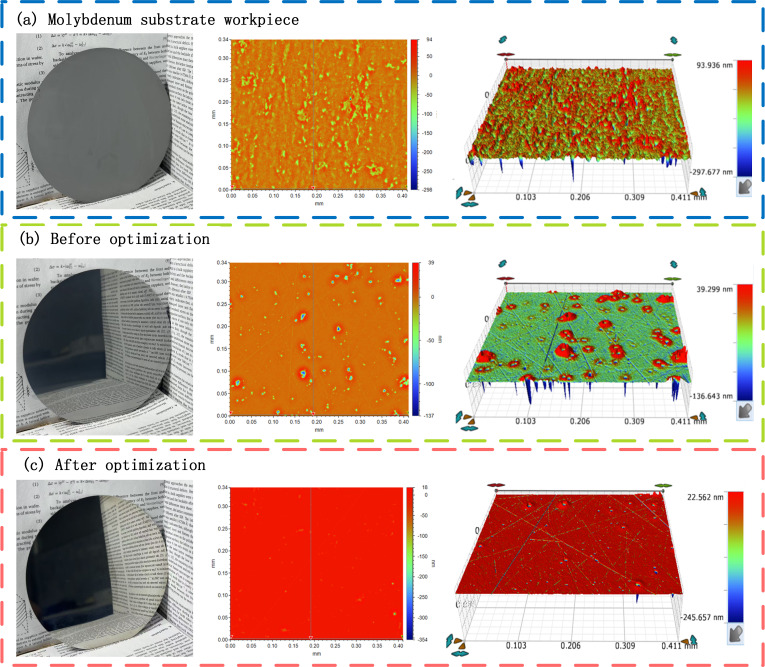
Molybdenum substrate workpiece and surface morphology.

Because the optimal parameter combination was not included in the *L*16 orthogonal experimental design, a verification experiment was conducted to confirm its effectiveness. In this verification test, double-sided CMP was performed on a molybdenum substrate under the following conditions: a polishing pressure of 115 kPa, a polishing solution pH of 11, H_2_O_2_ (0.5%) and glycine (5 mg/L) as additives, and an abrasive particle size of 0.6 μm. Figure 7 presents the molybdenum workpiece and corresponding surface morphology: (a) the molybdenum substrate workpiece, (b) the surface condition before optimization, and (c) the surface condition after optimization. The verification results indicate that the optimized parameter combination produced a higher surface finish following polishing, achieving an MRR of 80 nm·min^−1^ and a surface roughness Sa of 1.1 nm. Relative to the orthogonal experimental outcomes obtained prior to optimization, this parameter set not only increased the MRR but also further reduced Sa, demonstrating a clear improvement in both material removal efficiency and surface quality under the optimized conditions.

## 4. Conclusions

To improve both processing efficiency and surface quality in double-sided chemical mechanical polishing (CMP) of molybdenum substrates, an *L*16 orthogonal experimental design was employed to investigate the effects of polishing pressure, polishing slurry pH, additive system, and abrasive particle size on the material removal rate (MRR) and surface roughness (Sa). Multi-objective parameter optimization and validation were conducted using range/variance analysis and the weight-matrix method. The results provide practical guidance for balancing a high MRR with a nanoscale surface finish, thereby supporting the industrial-scale manufacturing of molybdenum substrates for LED applications. The main conclusions are as follows:

(1) The overall influence of the four factors shows that the additive system (*C*) is the dominant factor, followed by polishing pressure (*A*), with pH (*B*) and abrasive particle size (*D*) playing secondary roles. Specifically, for the MRR, the ranking is *C* > *A* ≫ *D* > *B*, while for Sa, it is *C* ≫ *D* > *A* > *B*. Thus, additives consistently exert the strongest effect; pressure is generally the second most influential factor; and pH shows the weakest main effect. In addition, Sa is more sensitive to abrasive particle size than to polishing pressure.
(2) Using H_2_O_2_ alone markedly increases the MRR but tends to increase Sa and induce pit defects. Introducing glycine suppresses excessive reactions, significantly reduces Sa, and decreases the likelihood of pits. Increasing polishing pressure generally enhances the MRR; however, excessively high pressure and larger abrasive grains raise local contact stress and indentation effects, thereby increasing the risk of indentation-type “pits”. Therefore, the H_2_O_2_–glycine system combined with moderate-to-high pressure can maintain a high MRR while achieving low defects and low Sa.
(3) The surface quality after double-sided CMP is governed by the coupled effects of process parameters. Based on multi-objective comprehensive optimization using the weight-matrix approach, the optimal parameter combination for the present system was determined as follows: a polishing pressure of 115 kPa, pH 11, H_2_O_2_ (0.5%) and glycine (5 mg/L) as additives, and an abrasive particle size of 0.6 μm. Under these conditions, a mirror-finish surface was obtained with an MRR of 80 nm·min^−1^ and Sa of 1.1 nm, meeting the processing requirements for high-quality, low-defect molybdenum substrate surfaces for LED applications.

## Figures and Tables

**Table 1:** The properties of the molybdenum wafer used in the experiment.

Lattice Constant/nm	Density *ρ*/(kg/m^3^)	Vickers Hardness (HV)	Thermal Conductivity/W·m^−1^ K^−1^	Poisson’s Ratio	Elastic Modulus/Gpa
0.315	10.2 × 10^3^	240–280	138	0.324	330

**Table 2:** The polishing conditions.

Factors	Parameters	Levels			
		1	2	3	4
*A*	Pressure [kPa]	64	77	90	115
*B*	pH	4	7	9	11
*C*	Additive	none	H_2_O_2_	H_2_O_2_ (0.5%) + Glycine (5 mg/L)	H_2_O_2_ (1%) + Glycine (5 mg/L)
*D*	Abrasive particle size [μm]	0.6	1.7		

**Table 3:** *L*16 (4^3^ × 2^6^) orthogonal test plan and results.

Factors	Parameters				Responses		
	Polishing Pressure [kPa]	pH	Additive	Abrasive Particle Size [μm]	MRR [nm/min]	Sa [nm]	SD
1	1	1	1	1	11	9.3	Pits
2	1	2	2	1	94	8.7	Pits
3	1	3	3	2	18	6.6	OK
4	1	4	4	2	20	7.8	OK
5	2	1	2	2	125	13.2	Pits
6	2	2	1	2	43	6.4	Pits
7	2	3	4	1	50	2.1	OK
8	2	4	3	1	57	1.6	OK
9	3	1	3	1	41	1.5	OK
10	3	2	4	1	18	4.3	Pits
11	3	3	1	2	61	9.6	Pits
12	3	4	2	2	144	9.9	Pits
13	4	1	4	2	131	2.3	Pits
14	4	2	3	2	88	2	Pits
15	4	3	2	1	156	10.5	Pits
16	4	4	1	1	51	1.4	Pits

**Table 4:** The influence of various factors on the MRR.

Factor	Freedom	Deviation	Mean Square Deviation	Test Result (F-Value)	Contribution (%)
*A*	3	10,082.5	3360.83	6.70	28.92%
*B*	3	551.5	183.83	0.36	1.58%
*C*	3	19,894.5	6631.5	13.22	57.07%
*D*	1	1444	1444	2.88	12.43%
*E* (error)	5	2506.5	501.3		

**Table 5:** The influence of various factors on Sa.

Factor	Freedom	Deviation	Mean Square Deviation	Test Result (F-Value)	Contribution (%)
*A*	3	35.21	11.76	2.02	16.14%
*B*	3	9.71	3.24	0.56	4.45%
*C*	3	109.83	36.61	6.31	50.35%
*D*	1	21.13	21.13	3.64	29.05%
*E* (error)	5	28.99	5.8		

## Data Availability

The original contributions presented in the study are included in the article. Further inquiries can be directed to the corresponding authors.
